# Understanding the barriers to NET-ZERO transport for rural roads: a Northern Ireland case study

**DOI:** 10.1186/s43065-021-00038-x

**Published:** 2021-09-13

**Authors:** Myra Lydon, Darragh Lydon, Nicola-Ann Stevens, Su Taylor, Juliana Early, Adele Marshall

**Affiliations:** 1 School of Natural and Built Environment, Queens University Belfast, Belfast, BT9 5AG UK; 2 School of Mechanical and Aerospace Engineering, Queens University Belfast, Belfast, BT9 5AG UK; 3 School of Mathematics and Physics, Queens University Belfast, Belfast, BT9 5AG UK

**Keywords:** Net-zero transport, Resilience, Transport networks

## Abstract

**Introduction:**

Climate-related disasters have cost the world over £450 billion over the last 3 years. In the race to mitigate these effects, the UK government has committed to net-zero emissions by 2050. Transport provides the largest single sector contribution to CO_2_ emissions, the road network accounts for up to 91%. As the only UK country without a formal climate change bill Northern Ireland could compromise the overall effort.

**Case description:**

In this research a survey of road asset owners, managers, academics, consultants, public transport providers was undertaken to seek to understand the current barriers to adapting a dispersed rural road network in Northern Ireland for net-zero transport. The survey data was collected though an online form with a combination of multiple choice and open ended questions. Thematic analysis was used to code and analyse the data collected which enabled a discussion around the key expert opinions gathered.

**Discussion and evaluation:**

The paper presents details of the current road network in Northern Ireland and highlights some of the issues faced by asset owners. The survey questions were developed though engagement with transport professionals in Northern Ireland and focus predominantly on road use rather than the impact of current land management practices or environmental conditions such as flood risk. The response highlights a clear enthusiasm for change in the operation of the public road network which is hindered by a lack of government strategy and limited public consultation.

**Conclusions:**

The high response rate (41%) for the survey highlights the interest of those in the transport sector to engage in activities which can support a better understanding of how road networks contribute to CO_2_ emissions. Within the survey data a requirement for behavioural change was highlighted as a key step to reduce transport related emissions, the enthusiasm for change demonstrates this is the optimum time to engage with the public and develop clear transport strategies. More accurate findings and empirical evidence could have been established had the study considered specific, transport planning, environmental and land use conditions for Northern Ireland. This will be the focus of further research in this area to enable clear translation of the research to other countries.

## Introduction

Climate-related disasters have cost the world more than £450 billion over the last 3 years [1]. In 2019, UK parliament passed legislation requiring a 100% reduction in net emission of greenhouse gasses relative to 1990 levels by 2050 [2]. This ambition requires an equilibrium between the amount of greenhouse gasses produced and the amount removed from the atmosphere, through carbon sinks such as oceans and forestry and carbon capture initiatives. Although it is unclear how it will be enforced, the climate action plan poses a legal obligation on government to address these issues. These commitments are becoming increasingly evident in planning decisions, in February 2020, the Heathrow airport expansion plans were deemed unlawful due to the adverse environmental risks [3].

The Climate Act 2008 requires UK government to set 5 yearly carbon budgets until 2031.While current levels are set to meet the third budget (2018–22) the UK is not on track to meet the 4^th^ and 5^th^ carbon budget [4]. The UK Nationally Determined Contribution commits to at least a 68% reduction by 2030. If achieved, this will be the fastest rate for a major economy to date [5]. The plan draws on several polices including the Ten Point Plan, Clean Growth Strategy and the forthcoming Net-Zero strategy [6, 7]. However, Northern Ireland as the only UK country without a formal climate change bill could potentially compromise the overall effort.

Transport provides the largest single sector contribution to CO_2_ emissions, and the road network accounts for up to 91% of this. Achieving targets will require a rethink of vehicles and infrastructure together. Therefore the future management and adaption of NIs most valuable asset (£26bn), the road network, needs to be a core component of climate change legislation in NI.

The route to net-zero transport is two-fold: reduce emissions and increase resilience to avoid the risk of disruption. Compared to development of low carbon emission technologies, the adaption of existing infrastructure to increase its ability to absorb and recover the effects of climate change is relatively unexplored [8]. During the last decade climate change adaption has been the focus of a significant body of research; however a gap remains in establishing adaption measures which are specific to the transport sector [9].

In this work, the NI road network is studied to understand the potential impact of condition and funding constraints on the resilience of a substantial UK road network and its ability to respond to climate change targets. While the NI road network is used as a case study in this research, it is the belief of the authors that the challenges faced will be representative of those faced by many global communities. Based on the current status of the network, a number of climate adaptation measures are explored. A survey of road asset owners, managers, academics, consultants, and public transport providers was undertaken to gauge the feasibility and potential barriers to adoption of the suggested measures. We disclose existing and potential issues relating to the current investment strategies and provide thought provoking scenarios for consideration when rationally developing adaption plans to mitigate the impacts of climate change and reduce carbon emissions associated with road networks.

## Case description

The paper is structured to provide a review of the current recommendations on net-zero goals followed by an evaluation of the current status of the NI road network. Review of the current status of net zero and NI roads section introduces the research methodology by detailing the scope of the open-ended survey. In this research a survey of road asset owners, managers, academics, consultants, public transport providers was undertaken to seek to understand the current barriers to adapting a dispersed rural road network in Northern Ireland for net-zero transport. Participant selection was chosen to be representative of those involved or who have a professional interest in the NI road network. The following organizations were contacted by email and invited to take part in the on-line survey:

- DfI- responsible for management and delivery of majority of NI road network
- Translink- responsible for public transport in NI
- Amey Consulting- heavily involved in the inspection and assessment of NI road network
- NI regional committee for Chartered Institution of Highways & Transportation – Sector Experts
- NI Institution of Civil Engineers- Sector Experts
- Academics actively researching in the area from Queens University Belfast

The methodology section provides the survey content and analysis of the responses including key quotations for the 8 topics included in the survey. The survey data was collected though an online form with a combination of multiple choice and open ended questions. Thematic analysis was used to code and analyse the data collected which enabled a discussion around the key expert opinions gathered. A brief literature review is provided with each topic to provide the reader with background knowledge of the proposed adaptation measure. The paper concludes with a general discussion and key recommendations for consideration by key stakeholders and researchers.

## Review of the current status of net zero and NI roads

The United Nations Sustainable Development Goals (SDGs) provides a blueprint for human equality and prosperity while protecting our planet’s ecosystems. A report by the United Nations Office for Project Services reported that infrastructure forms a central component in tackling all 17 SDGs and has a direct influence on over 70% of the specific targets that sit below them. Despite its underpinning importance, the true impacts of infrastructure and a networked delivery approach in achieving sustainability remains underexplored and underexploited in practice [10].

Legacy systems in the developed world, such as transport networks, are critical for a functioning society yet often sit in isolation of other infrastructure systems [11]. This limits the capacity of asset owners to fully transition the transport sector to net-zero emissions from its current position as single largest source of carbon dioxide emissions in the UK [12]. Reaching net-zero will require significant enhancement of shared infrastructure networks and a detailed understanding of the interdependencies and investment trade-offs which will enable a coherent overall strategy [6]. The road network is a vital element in the decarbonizing of the transport sector as passenger cars account for over 50% of transport emissions [13].

### NI road network

In 2018, UK roads had the second highest rate of congestion in the European Union, NI is more dependent on road transport than any other region in the UK, NI roads transport almost all freight compared with 76% in Great Britain (GB), at 84% NI has the highest rate of working population travelling by car or van in UK or Ireland. Rural roads make up 78% of the network and in many cases there is no practical alternative to private car use. NI has one of the densest road networks in Europe, measured by km or road per 1,000 inhabitants, NI is more than double the UK average and the rural population in NI is also almost double the UK average. Unlike any other UK country the management of the strategic, national and rural road network within NI falls under the responsibility of a single organization, the Department for Infrastructure (DfI). This structure provides greater opportunity to meet the recommendations outlined above and develop a connected transport strategy which supports net-zero goals. DfI is responsible for the management, maintenance and development of all public roads in Northern Ireland. Totalling approximately 26,000 km along with 10,000 km of footways, over 6000 bridges and approximately 370 public car parks, it has an estimated value of £26bn and stands as NI’s most valuable asset. The road network development strategies should reflect the need to facilitate safe movement of people, goods and services in a sustainable way that realises social and economic benefits.

In 2018, a detailed review of the condition of the NI road network was published by Barton which highlighted the impact of long term underinvestment [14]. Independently the Northern Ireland Audit Office recommended that on average an additional £51 million in annual funding is required to secure a sustainable road network [15]. Barton observed that annual costs for roads which are closely maintained to optimum condition are less than those with inefficient maintenance approaches. Late intervention typically costs up to four times the optimum [14].

Both reports found that in recent years DfI have made significant improvements in the day-to-day maintenance of the network, but these efficiencies have been outweighed by long-term financial pressures. This ultimately impacts the structural maintenance of the road network and has led to further deterioration in its overall condition. However, there has been a gradual improvement in the trunk roads and motorways in the last decade. The other roads making up the local road network have continued to deteriorate at a faster rate, as less money is made available to maintain them to the same standard. In 1998 it was estimated that £168 million (at today’s value) was required to clear the maintenance backlog; DfI have estimated the current cost today is £1.2billion [15]. This significant investment combined with the financial constraints is likely to see a continuing escalation of the deficit between required investment and available financing. There is a need for a fact-based future approach to allocation of funding for structural maintenance to ensure fair coverage for all sections of the network, including rural roads. Recent innovative approaches including DfI’s “Digital Roads SBRI Funding” show the promise of a forward looking approach to addressing the issues and highlighting the importance of data in the future of infrastructure planning [16].

### Northern Ireland road network bridge condition and management

Bridges are critical to movement of people, creating connections, unlocking economic opportunity, and providing access to health education and employment. Despite this, inadequate and varying budgets paired with the high capital cost of bridges results in limited redundancy in the provision of safe resilient structures. This exposes transport networks to fragilities at locations whereby an individual bridge provides the only means of utilities, passengers and freight crossing an obstacle meaning failure leads catastrophic consequences such as those witnessed in Cumbria in 2009 [17]. The probability of repeated events in the future is extremely high given that 40% of UK bridges are considered historic assets and the majority of our bridges possess similar attributes identified in the Cumbria failures [18]. DfI have collected a wide range of data from the inspection and maintenance of 6978 bridges, over several years. The current database stores all the current bridge inspection data following the Bridge Condition Index (BCI) rating system which was introduced on a phased basis. All legacy inspections condition rating, which utilized a 1–4 scoring method, is also held on the system.

### Investment in bridge maintenance

NI has the lowest investment in transport infrastructure per capita in the UK resulting in high vulnerability with lower capacity to respond to risks [10]. Funding for structures falls into two categories: Capital and Revenue. Infrastructure projects, major maintenance and improvements/changes to existing infrastructure comes under the former and routine maintenance and reactive maintenance comes under the latter. This analysis focuses only on the investment in the maintenance of bridges during the time period 2000–2020. From 2000 to 2014 there was a relatively stable investment of £2.9 million on average per year, subsequent funding commitment is shown in Table 1.

**Table 1:** Annual investment in bridge maintenance NI

Year	Spend (£million)	Year	Spend (£million)
2014/15	.54	2018/19	.81
2015/16	.46	2019/20	.80
2016/17	.81	2020/21	1.40
2017/18	.60		

An estimated valuation of £4.3bn has been made for bridges within the network. Only bridges with condition data have been included in the valuation, totalling 6047 bridges. The remaining structures do not meet the minimum criteria for inspection under requirements set out in CS 450 Inspections of Highway Structures [19]. The valuation has been estimated using the SAVI structures valuation tool in accordance with The Chartered Institute of Public Finance and Accountancy Code of Practice for the Highways Network Asset (2016) [20]. The structures are valued in terms of gross replacement cost. It is the starting point for calculating the net current value of the Highways Network Asset and its components – that is, their value after taking account of physical deterioration and all forms of obsolescence and optimization. Work is currently underway to estimate the true cost associated with the maintenance backlog, to understand how this compares with the £6.7bn backlog in Great Britain. As with the majority of transport asset owners, bridge maintenance is currently carried out on a reactive basis due to the current levels of underinvestment. This reduces the future resilience of the transport networks limiting the ability to prepare for extreme climate events which will in turn have negative impact on the route to net zero transport emissions. The “Growing back better” inquiry undertaken by the UK government in 2021 highlighted the need for a reform in the current road investment strategies. One of the recommendations was the introduction of a sustainability test to judge future investment decisions. Asset owners across the UK have developed ambitious digital strategies to transform the management of road networks. While the adoption of digital technologies will enable a data driven approach to future investment and provide an understanding of the investment trade-off, it will not provide the singular solution to achieving net-zero transport emissions by 2050. In this paper, the role of infrastructure investment in achieving net-zero transport ambitions is discussed, alongside an evaluation of the feasibility of a number of potential infrastructure adaptation measures to support transport decarbonisation..

## Methodology

### Open-ended survey

This study invited respondents engaged with the management & operation of the road network alongside academics engaged in research in the area to participate in an open- ended survey to explore the attitudes and views on future roadmaps to net zero in road networks. The survey respondents were chosen to be representative of those who have a professional interest in the road network in Northern Ireland based on an initial consultation with a Principal Professional and Technology Officer from DfI roads section. The participants have been assigned into three groups:

- **G1—Public:** State or semi-state organizations responsible for the delivery, maintenance and operation of the public road network and provision of public transport in NI.
- **G2—Academic:** Individuals actively researching in the area of highway infrastructure or low carbon transport in NI.
- **G3—Private:** Charities, privately owned organizations or independent consultants directly involved in the delivery, maintenance and operation of the public road network and provision of public transport in NI.

Throughout this paper quotations from those interviewed are presented and referenced using the notation in Table 2 (e.g. G1). The survey was anonymous and all opinions expressed in this survey are the personal opinion of the participant in a role directly related to road transport in Northern Ireland, not an official response of any organization nor do they necessarily represent the opinions of the organization. The survey was designed to provide an open snapshot of perceptions of proposed solutions to achieve net-zero transport at this moment in time.

**Table 2:** Notation for respondent groups

Group Description	Group Code	Position Range	% of Respondents
Public	G1	Deputy Secretary-Engineer	50%
Academic	G2	Professor-Researcher	23%
Private	G3	Director-Engineer	23%

Many alternative solutions to road network management have been proposed which could support the transition to net-zero transport emissions. To date there has been limited clear evidence of success from these proposals. Some may have the potential to elicit adverse initial public response. This research aimed to capture the personal opinions of individuals on the status, potential solutions, and barriers to net-zero transport emissions in NI. The open-ended survey was extended to those with professional experience of the NI road network and received a 41% response rate one individual refused to take part in the survey and the remainder did not respond to the invitation, the findings included are based on the opinions of 49 participants. The survey was distributed via email with embedded link to an online form to collect responses. The responses were then exported from the portal and analysed using NVivo [21]. Quantitative data was collected on 8 topics and in each case the respondent was given the option to elaborate on their response. This option had a 90% uptake rate. Content analysis was used to categorize, tag and enable thematic analysis of the qualitative data.

## Survey data

The questions and results of this analysis are presented in this section.

### Topic 1: current status of net-zero ambition

In June 2019 the UK became the first major economy to commit to net-zero transport emissions by 2050. Reaching this ambitious goal will require a dramatic shift across the delivery and use patterns of all transport infrastructure. More than ever, good governance and a coherent strategy are required to ensure the transition results in reduced exposure to climate risks and improved physical and mental health benefits for people. The initial signs for meeting the target are not positive. A report by The Climate Change Committee (CCC) found policy actions “[fell] well short of those required for the net-zero target” and the achieving net-zero was “technically feasible but highly challenging” [22]. The Survey asked:

1. “*Do you think the UK is on track to meet net-zero transport emissions by 2050?”*
2. “*Do you think the NI is on track to meet net-zero transport emissions by 2050?*

**Fig. 1:**
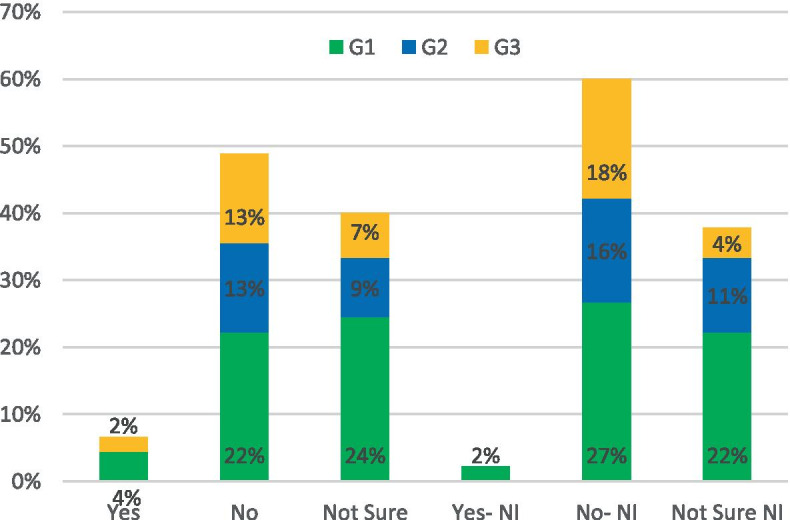
Response to: Do you think the UK/NI is on track to meet net zero emissions by 2050?

The opinions in terms of UK and NI readiness to meet net-zero transport are presented in Fig. 1. Overall, the predominant opinion was not hopeful, with only 7% of respondents believing the UK was on target and this reduced to 2% for NI. Positive responses anticipated that future innovations and enhanced efforts would increase the pace of change as we approach the deadline. There was agreement from 50% of respondents that the current UK government was not committed to reaching the target and the policy development was too slow and unclear. This is reflected in the response from G1 which emphasised a disconnect between those who set the targets and those responsible for meeting them: *“The government which made the commitments will not be in office to answer for delivery*”. More specifically “*NI is the only region in the UK without a specific climate change bill and has made the slowest levels of reductions since 1990. Whilst there are discussions ongoing on the creation of a bill without significant political leadership in the area it would be extremely unlikely*” was returned by a G2 respondent. After government commitment, the main concern was enabling the behavioural shift to meet the targets which was hampered by underinvestment in the infrastructure which would support the transition in both the UK and NI. The rural and dispersed nature of the NI population adds complexity to the issue. In both cases 12% of respondents acknowledged a lack of information on the topic. “*Even I who may be more likely (to have direct impact) am not aware of the measures and if they are likely to help the UK meet the target”*. (G1)

### Topic 2: investment in new road infrastructure

In 2020 UK central government pledged to spend £27bn on road infrastructure under the 5-year Road Investment Strategy 2 (RIS2) plan. The plan includes the construction of a number of new road schemes to “keep people and goods moving” [23]. Contradictory evidence to this statement has been published in 2019 report by Highways England which reviews a £317 m “pinch point” investment scheme. The review indicated that construction of new roads relieved peak congestion and improved the worst performing sections but has increased congestion in other areas. In general the increase was during the non-peak periods and led to a net dis-benefit over a 24 h period [24]. Recent research has indicated that RIS2 will add 20 million tonnes of carbon dioxide to UK emissions between now and 2032 and the construction of new roads within the scheme will negate 80% of the benefit gained from a switch to electric vehicles [25]. A Department for Transport (DfT) commissioned report found that in most cases, a 10% increase in capacity would result in at least 5% induced traffic. Case study evidence in the report highlighted that traffic increases on new routes were not offset by reductions on equivalent unimproved routes [26]. Overall, there is a question over the suitability of capital investment in new roads to reduce congestion and associated emissions. The implications of the court ruling on the proposed extension at Heathrow has the potential to affect RIS2 on the grounds that it is inconsistent with the legally binding climate change commitments [3]. Appropriate econometric analysis based on UK data would provide a useful addition to the evidence base which would in turn enable a better understanding of the carbon consequence before embarking on a long-term road building strategy.

The survey asked: *Do you think new road infrastructure should be built to reduce congestion?*

**Fig. 2:**
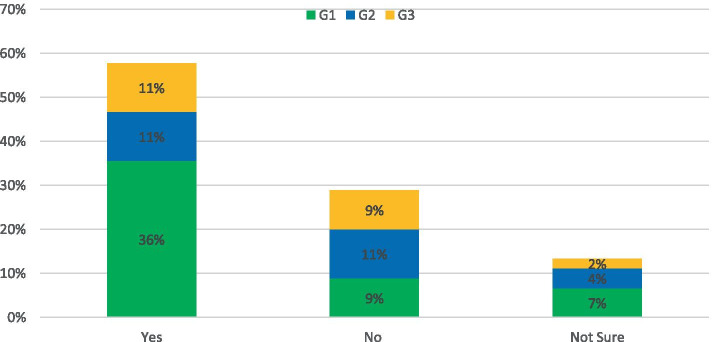
Response to: Do you think new road infrastructure should be built to reduce congestion?

For this topic, the response was: 58% of respondents said yes, 23% of respondents said No and 19% were not sure if new roads would aid the congestion problem currently in evidence in NI (Fig. 2). While a substantial portion of responders did believe that investment was necessary, this did not necessarily take the form of new routes as such. Rather they were concerned about the poor condition/capacity of existing roads to support expanded public transport services and the belief was that investment was key to ensure a greener transport network in the future. The respondents that did not support additional investment believed that making it easier for car journeys to take place would be self-defeating, and that funds marked for road development could potentially instead be used to re-establish the rail network in NI. This was summarized by the following response: “*There is a limit to how far road infrastructure on its own can contribute to reducing congestion. A modal-shift (supported by appropriate infrastructure) can potentially have a greater effect than a new road from the perspective of journey-time.”* (G1)

### Topic 3: de-adoption of roads

The economic impacts of climate change on road infrastructure in terms of high cost for adaption, maintenance and negative impact on transit have been widely researched [27, 28]. A gap remains between isolating the assessment of impacts and tangible actionable results that inform decision makers on the key investment areas to mitigate and adapt to the climate change impacts. Schweikert et al. [29] analysed the impacts of both extreme events and incremental climatic changes on road infrastructure in 10 geographically and economically diverse countries through to 2100. 54 distinct AR4 Global Circulation Model scenarios of future climate change were tested to compare the outcome of a reactive and pro-active adaption measures. Within the study, Italy provides the closest representation of the UK, in terms of land area, World Bank defined income level, total road length and GDP. The 2014 findings are output in “opportunity” and “regret costs” for adapt (proactive) or no adapt (reactive) options. “Opportunity” represents the amount of future infrastructure development which will not occur because money is now being redirected to climate change related costs. “Adapt regret” is the amount of money lost if a proactive approach is taken and climate change does not happen. “No adapt regret” is when a reactive approach was taken and climate change occurred as predicted in the model. By 2010 the median opportunity cost with adapt policy was calculated at 18% increasing to 34% for a no adapt policy. In the same decade a median “apart regret” cost of $1,087 m was calculated compared to $20,032 m for “no adapt regret” cost. In each country included in the study, a proactive adaption is less costly than a reactive no adapt policy. The inclusion of climate modelling tools similar to the US —Climate Change Adaptation Tool for Transportation can inform future policy for road infrastructure investment in the UK. This would enable critical roads located in high impact Climate Research Unit grids to be prioritized for investment. This approach will enable the early identification of routes which may not be sustainable to maintain within the network. The imminent threat of climate change in the UK has initiated the development of a council led task force to relocate the residents of Fairbourne in North Wales before 2050 due to rising sea levels. NI has one of the densest public road networks in these islands and a very dispersed rural population. This coupled with long term underfunding and increasing extreme climate events compromises the overall sustainability of the road network. It is the authors’ opinion that a future reduction in the NI road network should not be ruled out and an early understanding of the public participation in such a scheme would better prepare the country for climate change.

The survey asked: *Do you think parts of the road network could be un-adopted, repurposed or privatized as a feasible solution?*

**Fig. 3:**
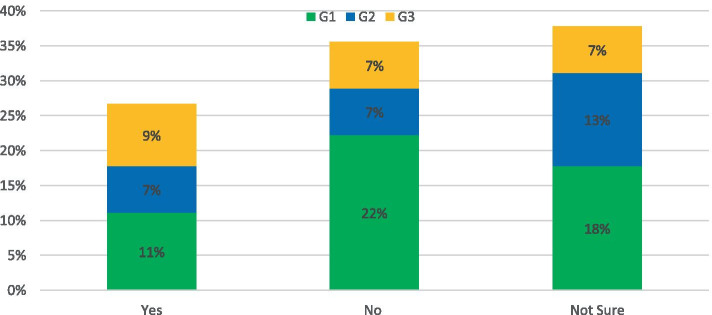
Response to: Do you think parts of the road network could be un-adopted, repurposed or privatized as a feasible solution?

As shown in Fig. 3, the responses for this topic were cautious at best, negative at worst. While 25% of respondents were in favour of the proposed solution, 90% of that total indicated they did not think it would be feasible to do so. The remainder of the answers were mainly concerned that such an approach would not be practical to implement.

The survey asked: *Would you support a pilot scheme whereby a road/bridge is selected for potential decommissioning for motorized vehicles to enable better walking/cycling infrastructure or as a community asset transfer?*

The response to this topic was highly positive, albeit with the caveat that this should only be implemented if a suitable site was available. The need to determine a suitable site was primarily due to concerns that the local community would object to this project or that closing a bridge would lead to traffic migration into other areas, leading to increased pollution/congestion in those parts of the network.

### Topic 4: congestion/toll charging in Northern Ireland

Congestion pricing or toll charges are considered one of the most efficient solutions to combat congestion and provide an associated reduction in carbon emissions. They are introduced by charging a fee for use of a certain road to reduce traffic demand or to distribute the traffic demand more evenly among the traffic network. One of the most widely used methods for introduction of a charge is through a toll cordon which is a form of area-based charging in which vehicles pay a toll to cross a cordon in the inbound direction, outbound direction or possibly in both directions. Several major cities across the world including London and Singapore have adopted this type of congestion charge. Following the introduction of the scheme in London, preliminary results were published in 2004 [30] which showed that in the first year of the scheme, congestion has decreased by 30%. However, there have also been many unsuccessful attempts [31] in cities such as Manchester, Copenhagen, Edinburgh and New York [32].

The set-up and operating costs play a significant role in deciding if the introduction of the toll is financially viable. In the first 2 years of the scheme being introduced in London, costs were reported as £95 million which was more than twice the cost that was predicted [33]. In addition to the predictions about the costs being inaccurate, the expected revenue fell short of expectation. This reduction in the expected revenue was a result of the scheme being more effective than anticipated in deterring people from driving into the centre of London in addition to the higher number of vehicles which were exempt from the charge or were entitled to reduction in the fee such as taxis or low emissions vehicles than originally anticipated. One of the reasons why the scheme was adopted was that the revenue generated from the scheme was for spending on public transport around London and a report in 2017 [34] estimates a total of approximately £1.7 billion income from the scheme since its introduction. There are a few key reasons why this congestion charge has been a success. Firstly, London’s public transport system functions well and provides potential road users with alternatives forms of transport around London. If NI were to enforce a congestion charge, careful consideration would need to be given to toll charge, how the toll will be collected and how it will be enforced.

Survey question: *Would you support a pilot scheme to charge for access to certain roads or urban centres?*

**Fig. 4:**
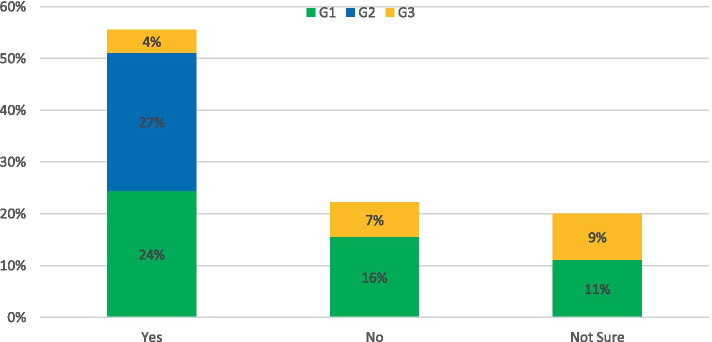
Response to: Would you support a pilot scheme to charge for access to certain roads or urban centres?

Figure 4 shows that the options of “Yes”, “No” or “Not Sure” received 56, 23 and 21% of votes respectively. When asked to comment on potential advantages or problems with the scheme the predominant concern across each group was public opposition and a resulting inequality in access to centralised job, health or education benefits. A recurring solution to this was transparency in the financial outcome of the scheme with clear reinvestment strategies in alternative transport solutions. The key perceived positive benefit was the reinvestment of financial gain from the scheme could enable alternative greener solutions for all. However, as detailed above the implementation of a city-based congestion based scheme in Northern Ireland would likely cause additional financial burden which would outweigh the benefits of the reduction in carbon emissions. Additionally as quoted by a G1 respondent *“Who would wish to take on such a burden?.* Alternatively, adding a toll cost to the strategic network would have significantly less financial burden at operator level while increasing the cost of everyday car use and perhaps incentivising road users toward alternative transport methods. The implementation of such a scheme would require a holistic approach to avoid increasing transport inequalities and provide transparent benefits to the public through reinvestment strategies.

### Topic 5: impact of COVID-19 on transport emissions

Lockdown restrictions due to COVID-19 have fundamentally changed the way people travel, work and live [35]. The reduction in passenger transport demand has had a widespread impact on the transport sector, with many major cites seeing 90% reduction in trips [36]. The long term effect of this temporary reduction in transport emissions is likely to be negligible, however the recovery presents a once in a life time opportunity to reset investment decisions in maintaining the transport network and providing accessible affordable public transport. The opportunity for disruption to act as a catalyst for a previously inconceivable shift change toward more sustainable transport has been seen on a smaller scale pre-COVID. In 2009 excessive flooding in Cumbria severed 4 of 5 river crossing points in the town of Workington, leaving only the rail crossing. This caused a modal shift in the travel behaviours and overall journeys within or around the town reduced by over a third. Interestingly, 7% of car trips taken pre-flood had made a permanent modal shift stimulating long term unanticipated carbon reduction benefits [37]. The disappearing traffic phenomenon was further evidenced by Cairns [38] confirming that the removal of some elements of the highway infrastructure does not necessarily relocate traffic away but can provide an successful means of reducing overall traffic levels in the longer term [38]. Examining past crises can be used to inform on policy which would incentivize positive behaviours and discourage the return to business as usual. After 9/11, US domestic travel was estimated to be 7% less 5 years after the attacks had happened compared to if they had not occurred [39]. Similarly, after the London bombing in 2005 a clear modal shift occurred and bike retailers reported a fourfold increase in bike sales in the weeks after the events. In both cases the increase of private vehicle use and move away from public transport post event due to so called “dread-hypothesis” resulting in and environmental benefits of positive behaviours’ being negated [40]. In a crisis travel behaviours unquestionably adapt in the short term to account for the disruption, the opportunity needs to be harnessed to embed this change in long term practice. As the economy is rebuilt post Covid it is vital that governments support positive behavioural shifts and the diffusion of information to shape distorted persecutions of risk to help achieve reductions in carbon emissions.

The survey asked: *Do you think COVID-19 provides an opportunity to reset NI’s transport emissions though better working from home (WFH) infrastructure?*

**Fig. 5:**
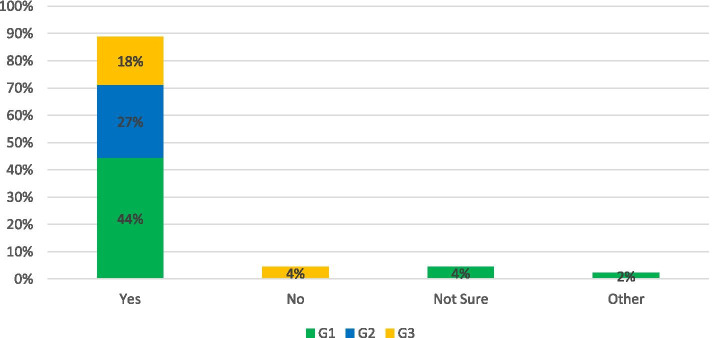
Response to: Do you think COVID-19 provides an opportunity to reset NI’s transport emissions though better WFH infrastructure?

The response to this question was nearly unanimous, with 88% of survey respondents answering Yes. This was consistent across all response groups, with 100% of academic respondents agreeing as can be seen in Fig. 5. An interesting theme to emerge from the responses was that Covid-19 could adversely affect public transport and result in an increase in single car occupancy journeys as stated by a G2 respondent: “*Anecdotally, I know of a number of people who would have always used public transport or carpooled who will now only use their private car to commute to work, and I would assume that this behaviour is not limited to a small number.”*

The survey asked: *What other opportunities has Covid-19 created to reduce emissions?*

Apart from the main theme of WFH changes to behaviour, the prevalent sentiment in this topic was that the lengthy lockdown will have given people in NI a vision of a greener future with a shift in prioritization of work-life balance. The concerns were twofold: 1) The shift to WFH will only be temporary and the public’s eagerness to return to the status quo will result in car journeys returning to pre-pandemic levels. 2) That WFH does not actually provide the reduction in emissions it has been widely assumed to have done. As stated by this G2 respondent: “*Office spaces are still being heated but working from home causes homes to be heated—this is a big emissions problem. We are potentially increasing emissions in the biggest area!”*

The survey asked: *What actions are needed to underpin this opportunity?*

While the vision of a greener future discussed in the previous question was viewed by respondents as a laudable ideal to work towards, there were some disagreement in the suggested actions to capitalize on the seismic shift presented by Covid-19. There were two schools of thought as to how to proceed with these actions. The first theorized that a comprehensive program of building greener infrastructure was necessary to enable the step change in transport habits for employees travelling to work. This mind-set was common across all categories of survey respondent. The second group, primarily public sector employees, believed the focus should be on employers to lead the change in public behaviour by facilitating remote working and making videoconferencing the preferred method of communication between members of a workforce. Both groups did agree that a shift in public behaviour would be necessary if either of these approaches were to be successful.

### Topic 6: prioritizing “greener above faster” transport strategy and maintaining transport equality

Mobility is central to all of society and an efficient transport network has been seen as the foundation of economic growth in the UK. Undoubtedly the next two decades will deliver unprecedented innovation to the transport sector both in terms of technology and user behaviour. Building capacity has previously been the main driver for transport strategies but as it seems less likely engineering innovation will enable net-zero transport there is a need to consider behavioural and social science approaches [41]. Under the UK net-zero ambition transport strategies must aim to create safe, green, healthy, connected and inclusive communities while enhancing economic growth. With almost 80% of roads in NI classed at rural there is a significant challenge in adopting a fully green transport strategy whilst maintaining the current level of service. It is expected that by 2040 people aged over 65 will become the largest population segment in rural areas; this coupled with the current trend of younger people travelling less is likely to have an impact in the future demand for commuter travel [42]. A potential solution would be to address this from a localization perspective, whereby communities can access work social and health facilities in their local area reducing the need to travel.

The survey asked: *Should transport strategy be aimed at connecting people in greener ways rather than faster?*

**Fig. 6:**
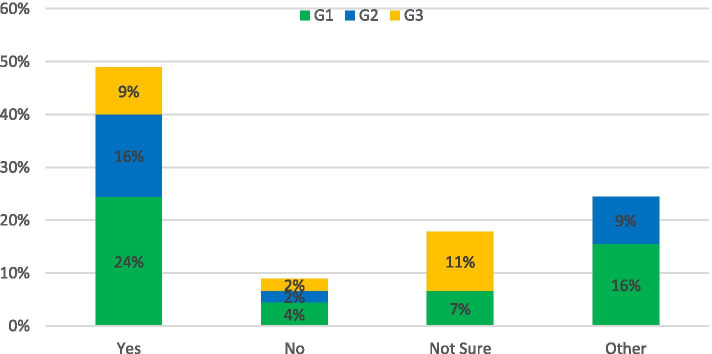
Response to: Should transport strategy be aimed at connecting people in greener ways rather than faster?

The majority of respondents to this survey did not believe that a heavily “green-focused” transport strategy would be a viable goal as they did not believe the two approaches should be mutually exclusive. The reasoning behind this was subtly different for responses from the G2 category compared to G1 and G3. Respondents from G2 tended to believe that technological improvements would solve this issue, while members of the public and private sector were clear in their belief that policy and the public mind-set would be more important considerations in the adaptation of a widespread greener infrastructure. This is reflected in the large percentage of respondents choosing the “other” option as presented in Fig. 6. A G1 member stated: “*I think transport strategies need to look harder at the psychology behind people’s choices and aim to change attitudes rather than get stuck developing engineering solutions to what are behavioural problems.”*

The question was followed up with: Do you think the public would support such a strategy?

**Fig. 7:**
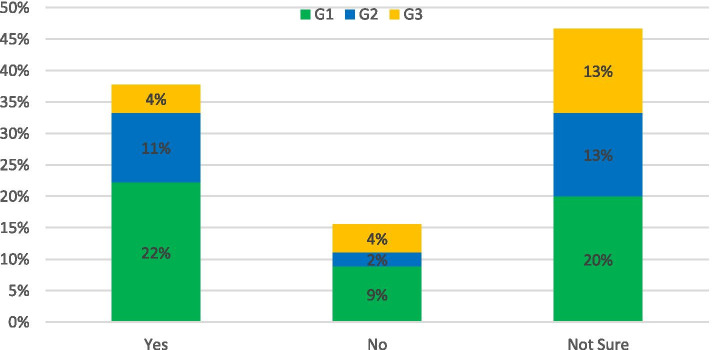
Response to: Public Support for Greener Transport Solutions

The problem of convincing the public about the benefits of green infrastructure is further developed in the answers to this question. Opinion was skewed slightly negatively amongst respondents, with only 36% believing the public would have unqualified support for this strategy. The results for each respondent category are shown in Fig. 7. While there is a widespread belief that the public will not support difficult change, this is not a negative an outlook as it appears at first glance. Numerous respondents stated that as long as the costs and benefits of the approach are made clear to the public, support will follow as there is widespread knowledge of the climate crisis amongst the population. There is also confidence that if suitable policy is set, the public will adhere to the new guidelines in a similar manner to previous public safety campaigns: “*Society is resilient and will change, examples include the attitude to seat belts and drink driving.”*(G1)

### Topic 7: ensuring transport equality for the population while reducing carbon emissions

Previous research has provided clear evidence that centralized infrastructure investment in urban areas (urban bias) leads to social and economic and digital poverty in less connected rural areas [43]. Over the last two decades there has been a clear and successful transport strategy to ensure all regions have access to education, hospitals, and work opportunities and public amenities. In NI this has resulted in the construction of flagship road projects such as A8 Belfast to Larne dual carriageway. As such, the private car has maintained its top position as the most cost and time effective method of travel outside urban areas. Public transport provides a clear benefit to the reduction of transport emissions but without adequate widespread service the prioritization of public transport investment over the structural maintenance of all roads creates an inequality to rural areas. In a culture so reliant on car use the economic cost of providing an underutilized public transport service to all rural areas could have detrimental effects on the sustainability of service in urban areas where demand is high. Recent studies examining private car use in other European countries has provided valuable insights, as such empirical evidence is needed on the behavioural reasoning for such extensive car use in the UK to inform on policy change [44]. A significant government investment is required to enable the behavioural shift required to reduce our car dependency and meet net-zero targets whilst maintaining equal opportunities to rural and urban communities. In the survey this topic was left intentionally broad to understand the respondents’ perception of transport inequality in NI.

The survey asked: *Can investment in in roads providing public transport be prioritized whilst still maintaining transport equality?*

**Fig. 8:**
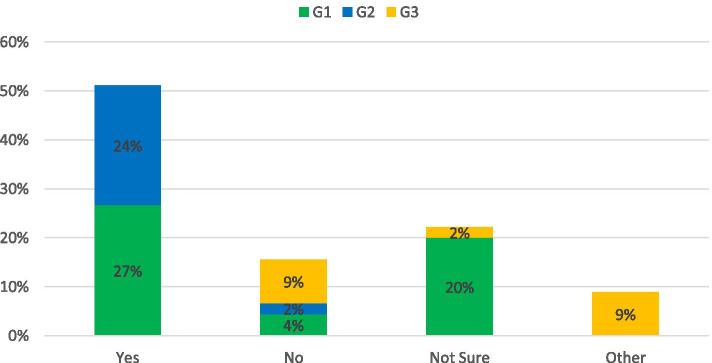
Response to: Can investment in in roads providing public transport be prioritized whilst still maintaining transport equality?

The response was encouraging with 59% of respondents agreeing and 31% unsure (this percentage is a combination of other and not sure categories in Fig. 8). There was a consensus that since public transport in NI is predominantly road based then investment in the network would have benefits to all users. The uncertainty was based on two emerging themes with a dominance on rural connectivity and concerns that low traffic routes which would be unsuitable for our traditional approach to public transport would not be maintained to an adequate level. Subsequently it was acknowledged that if car use remained the easiest modes of transport then demand will not be reduced. One G3 member suggested: *In the absence of sufficient volumes for high capacity transport a smaller demand responsive option is required.*

*Do you believe that there is a need to expand public transport services to eliminate/reduce transport inequality?*

**Fig. 9:**
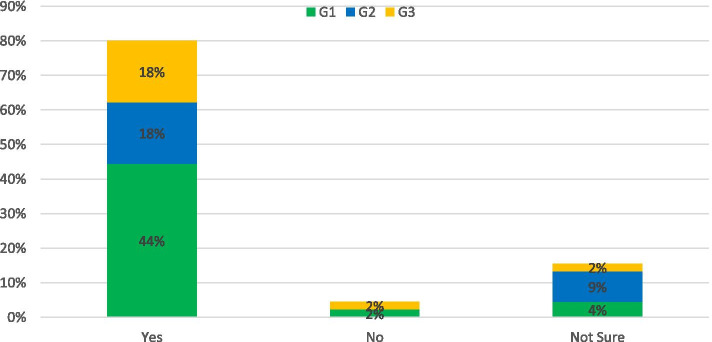
Response to: Do you believe that there is a need to expand public transport services to eliminate/reduce transport inequality?

A minority (4% of respondents), spilt evenly between G1 and G3, disagreed that there was a need to expand the public transport network in NI. However, where justification for this response was provided the opinion was based on the need to first make public transport more affordable to all through increased government support (G1). The cost and accessibility of public transport was echoed across G1-3, suggesting that even the most reliable and utilised services are not accessible to all. *The public transport model alienates families because it charges per person, whereas a private car is paid per vehicle (eg one parking space). Getting a Glider may be slightly cheaper than inner city parking, but not by much* (G2). Figure 9 shows that the overwhelming opinion of the 80% who agreed public transport needed to be expanded believed that rural areas are disadvantaged by the current public transport model citing: *If you don’t have a car it would be nearly impossible to live in our area.* (G3). Across all groups there is a pervasive feeling that the public transport in NI needs to be reviewed and expanded whist implementing a more “*flexible and responsive public transport services for regions where the demand overall may be lower or less predictable (G2).* A strategic multimodal structured approach which encompasses car sharing, park and ride, traditional public transport, shared taxis, cycling and walking was highlighted as the most feasible solution to reduce the dependency of single occupancy car use in rural areas. This integration of multiple forms of transport services into a single on demand user interface is known as “Mobility as a Service”. The biggest impact of this approach is the paradigm shift away from car ownership. Studies across European countries have been successful, for example in Sweden private car usage dropped 44% and in Vienna a 20% drop was recorded during a pilot scheme [45]. The potential of Mobility as a Service in rural dispersed population has been largely unexplored.

### Topic 8: harnessing contribution of public transport

As we move towards net-zero emissions, understanding the impact vehicles will have on our infrastructure will be of key importance to ensure sustainable services. The public transport sector with its regular service routes offers particular opportunities for added value. By stimulating a digital transformation in the monitoring, processing and analysis of information about our infrastructure, we can predict how it will perform under changing vehicle loads, cycles of loading and environmental factors, and move towards smart infrastructure. The cost of repairing faults as they approach criticality is enormous, but if damage is prevented at an early stage, alongside accurate risk modelling, earlier interventions can be made. Another challenge which has faced the low energy smart infrastructure is the requirement for a continuous power supply to structural health monitoring systems. Drive-by systems can negate this, but current research on drive-by monitoring systems with efficacy have not been fully demonstrated. The bus sector offers an interesting test case, with regular routes enabling continuous monitoring of critical infrastructure components. A drive-by inspection system should be capable of capturing damage by detecting changes in the mechanical properties. Drive by inspections could be used to inform on bridge properties, allowing for up to date information to be used in machine learning algorithms for prediction of future bridge condition.

The survey asked: *Do you believe that public transport data can support our public road network and provide data on road condition and usage?*

**Fig. 10:**
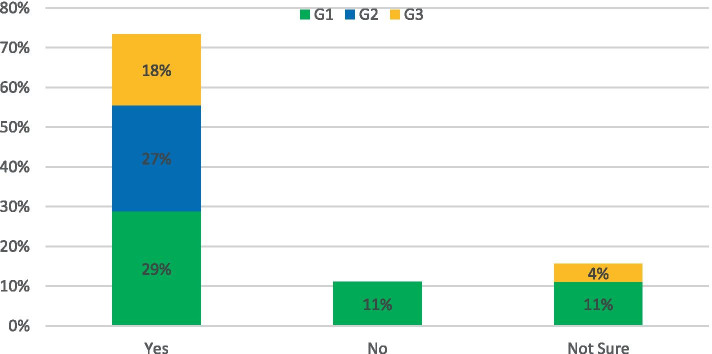
Response to: Do you believe that public transport data can support our public road network and provide data on road condition and usage?

73% of respondents believed that public transport data should be used to inform on the condition of road infrastructure. This was particularly evident in the academic cohort with 100% of respondents believing this was a viable solution. A concern raised by those who were not sure was that the data gathered from onboard monitoring would not be accurate enough to obtain valuable insights on infrastructure condition. Research is currently underway by the authors in this area in order to produce a solution that can be deployed across the road network. A G3 respondent pointed out that “*Operators will not always run the same vehicle on the same route on regular basis.”* It is the belief of the authors that a standardised method of data collection would remove the need to have the same vehicle consistently travel the same route. The responses for this topic are presented in Fig. 10.

## Discussion and conclusions

The findings from the survey of a diverse range of experts in this study offers an insight into the current position of NI to meet net-zero transport emissions. Overall the delay in policy development, funding constraints and the dependency on personal car use are considered to be the top three barriers to achieving net-zero followed by the rural nature of the NI road network. This is unlikely to be limited to NI, and the findings of this research provides insights for other global regions with highly dispersed populations. The financial constraints present in NI are also likely to be replicated in other regions worldwide. This means that the suggestions presented here would have scope for application in similarly sized nations or regions. The participants of the study were divided into three categories in terms of their sector connection to the road network, public, academic or private. Respondents from public organizations held the most concerns that underinvestment in public transport is causing significant delays on the road to net-zero.

There was almost unanimous agreement that NI is not on track to meet net-zero targets and a widespread lack of understanding of the consequences of a future carbon surplus. This suggests that in addition to an urgent need for policy change there is a requirement for an intense knowledge transfer campaign to ensure all stakeholders and the wider public have the information required to drive the paradigm shift away from single occupancy car journeys. Notably, a positive sentiment for the adoption of more climate friendly transport strategies was identified from all respondents. Although this was recorded at varying levels of enthusiasm it demonstrates the willingness of all parties to support strategies with benefits which will only be realised beyond the time horizons considered by current investment strategies.

The impact of the Covid -19 Crisis demonstrates the consequences of failing to adapt for future risks due to uncertainty. Moving forward from this crisis there is an opportunity to rethink the transport network and initiate new behaviours. Utilizing the Covid crisis, it is time to move forward with strategy development, accelerate the learning with transfer of information sector and dramatically scale up our efforts.

In the case of climate change, the risks are uncertain and unquantifiable which hinders the integration of a climate adaption plan. The uncertainty does not validate inaction, a systematic strategy is needed which can identify specific adaption measures for vulnerable assets such as those that make up the UK transport networks. This level of uncertainty is new and distinct from the risks engineers traditionally design for. Future approaches need to facilitate adaptive decision making which incorporates uncertainty rather than trying to engineer it out. There is a universally shared responsibility to develop solutions to not only reduce carbon output but also enhance the resilience of the networks to meet the inevitable consequences of climate change. Researchers and private organizations need to establish empirical evidence which can inform policy development to ensure efficacy in the outcomes. The race to net-zero gives the opportunity to embed climate resilience into our transport infrastructure.

Investment strategies like RIS2 provide a risk of over-engineering to attempt to reduce ambiguities. The Barton report in 2018 has provided a clear picture of the current condition of the NI road network and has acted as a catalyst for change to a systems approach for management of the network. The response to the survey carried out within this research illustrated the desire and willingness of the public, private and academic sectors in NI to come together and develop solutions for safer, greener and more resilient infrastructure. This pioneer survey of the NI road network will contribute to future more detailed feasibility studies of the solutions proposed along with increased public engagement to understand the implications of future decision on those who are particularly vulnerable. The results will be shared across all participants including highway authorities, public transport providers, transport consulting companies and governments. This will highlight the significance of net-zero on future transport strategies and a better indication of the potential uncomfortable truths facing network providers and stakeholders. These ideas will open further research questions to develop solutions to simultaneously net-zero targets and adopt networks to mitigate the risks of climate changes.

## Data Availability

Data available on request from the authors. Data Availability Statement (DAS) The data that supports the findings of this study is not openly available as the complete data set may enable the reader to identify participants through a combination of organisation and role data. To ensure participants remain anonymous sections of data including the complete quantitative data and response to open ended questions are available from the corresponding author upon reasonable request.

## Abbreviations

NI: Northern Ireland
UK: United Kingdom
GB: Great Britain
SDGs: Sustainable Development Goals
DfI: Department for Infrastructure
CCC: Climate Change Committee
RIS2: Road Investment Strategy 2
DfT: Department for Transport
WFH: Working from Home
